# The accuracy of the clinical diagnosis of Parkinson disease. The HUNT study

**DOI:** 10.1007/s00415-018-8969-6

**Published:** 2018-07-10

**Authors:** Eldbjørg Hustad, Anne Heidi Skogholt, Kristian Hveem, Jan O. Aasly

**Affiliations:** 1 Department of Neurology, Molde Hospital, Møre and Romsdal Hospital Trust, Molde, Norway; 2 Department of Neuromedicine and Movement Science (INB), Norwegian University of Science and Technology (NTNU), Trondheim, Norway; 3 Department of Public Health and Nursing (ISM), Norwegian University of Science and Technology (NTNU), Trondheim, Norway; 4 The Nord-Trøndelag Health Study, Norwegian University of Science and Technology (NTNU), Trondheim, Norway; 5 Department of Neurology, St. Olavs Hospital, 7030 Trondheim, Norway

**Keywords:** Parkinson disease, Diagnostic accuracy, Health registers, Biomarkers

## Abstract

Diagnostic accuracy is crucial not only for prognostic and therapeutic reasons, but also for epidemiologic studies. We aimed to study the accuracy of the clinical diagnosis of Parkinson disease (PD) for participants in The Nord-Trøndelag Health Study (HUNT), a health survey, containing data from approximately 126,000 individuals and biological material from 80,000 individuals. We included 980 participants from the HUNT study diagnosed with PD or secondary parkinsonism/related parkinsonian disorders. The participants had been diagnosed in conjunction with admission to hospitals in Trøndelag or through out-patient examination. We validated the diagnosis of PD by reviewing available Electronic Health Records (EHRs) using the MDS Clinical Diagnostic Criteria as gold standard. In total 61% (601/980) of the participants had available EHRs and were selected for validation. Out of those, 92% (550/601) had been diagnosed with PD while 8% (51/601) had been diagnosed with secondary parkinsonism/related parkinsonian disorders. The main outcome measure was the accuracy of the clinical diagnosis of PD for participants in the HUNT study. We verified PD in 65% (358/550) and excluded PD in 35% (192/550) of the participants. According to our results, the overall quality of the clinical diagnosis of PD for participants in the HUNT study is not optimal. Quality assurance of ICD codes entered into health registers is crucial before biological material obtained from these populations can be used in the search of new biomarkers for PD.

## Introduction

Parkinson disease (PD) is a progressive neurological disorder characterized by large numbers of motor and non-motor features which causes a heavy burden both on those affected, as well as their families. We mainly base the diagnosis of PD on clinical criteria. Definite diagnosis, however, are obtained only pathologically [1]. Correct diagnosis is crucial for prognostic and therapeutic reasons, but also for epidemiologic studies [2]. Population health data sets are helpful tools to evaluate health outcomes and health services as well as to describe the clinical course of disease and the total burden of disease and interventions in the population. They can provide data for the assessment of risk factors, causal pathways, positive and negative correlations with other diseases and rare outcomes in addition to provide accurate measures of disease, which, in turn, can define incidence and prevalence of a disease [3]. However, diagnosis-coding error is a challenge. According to O’Malley et al. [4], there are main sources of diagnostic coding error both along the ‘‘patient trajectory’’ (the dynamic interplay between the patient as he or she progresses through the health care system) and the ‘‘paper trail’’ (the creation of the medical record). Main sources of error along the ‘‘patient trajectory’’ include “the amount and quality of information at admission, communication among patients and providers, the clinician’s knowledge and experience with the illness and the clinician’s attention to detail. Main sources of error along the ‘‘paper trail’’ include “variance in the electronic and written records, coder training and experience, facility quality-control efforts and not intentional and intentional coder errors.

The Nord-Trøndelag Health Study (HUNT) was conducted in three waves of data gathering and biological sampling, the HUNT1 Survey (1984–1986), the HUNT2 Survey (1995–1997) and the HUNT3 Survey (2006–2008). Today, the HUNT study databank contains health survey data from approximately 126,000 individuals, both family data and individual data, which can be linked to national health registries. Biological material was collected from all participants (80,000) in the HUNT2 and HUNT3 surveys. The diagnosis of PD can be straightforward when patients have a classical presentation. However, differentiating PD from related parkinsonian disorders early in the course of the disease, when signs and symptoms overlap with other syndromes, may be challenging [5]. The aims of our study were to assess the accuracy of the clinical diagnosis of PD for participants in the HUNT study.

## Method

We identified all participants in the HUNT study who had been diagnosed with parkinsonism by comparing the eleven-digit unique personal identification number (PIN) of the participants who had taken part in at least one HUNT Survey to the official registry of PD diagnosis during the same period. The diagnoses were set at hospitals in Trøndelag. The following ICD codes were used: (ICD 10)/332 (ICD 9) (PD), G21 (secondary parkinsonism), G21.1 (drug-induced secondary parkinsonism), G21.2 (secondary parkinsonism due to other external causes), G 21.3 (post encephalitic parkinsonism), G21.8 (another secondary parkinsonism), G21.9 (unspecified secondary parkinsonism), G23 (other degenerative diseases of basal ganglia), G23.1 (progressive supra nuclear palsy), G23.9 (unspecified degenerative disease of the basal ganglia) and G25.8 (other special extrapyramidal conditions).

Cardinal signs of PD are asymmetric bradykinesia, rest tremor and rigidity. To differentiate PD from related parkinsonian disorders, we use the presence and specific presentation of the cardinal signs. Additional clinical features include secondary motor symptoms (hypomimia, dysarthria, dysphagia, sialorrhoea, micrographia, shuffling gait, festination, postural instability, freezing, dystonia, glabellar reflexes) and non-motor symptoms (autonomic dysfunction, cognitive/neurobehavioral abnormalities, sleep disorders and sensory abnormalities such as anosmia, paresthesias and pain) [6]. “Absence of rest tremor, early occurrence of gait difficulty, postural instability, dementia, hallucinations, and the presence of dysautonomia, ophthalmoparesis, ataxia and other atypical features, coupled with poor or no response to levodopa, suggest diagnoses other than PD” [6].

Since there is no definitive test for the diagnosis of PD, we based the diagnosis on the MDS Clinical Diagnostic Criteria for PD [7]. We reviewed Electronic Health Records (EHRs) of the participants in the HUNT study and classified them according to these criteria. If not seen by an experienced neurologist, the PD diagnosis were accepted only if the EHRs contained sufficient information on tremor, bradykinesia and rigidity. The long-term follow-up effect of levodopa was also emphasized.

We classified participants as probable PD if the EHRs described parkinsonism (defined as bradykinesia in combination with at least one of rest tremor or rigidity), combined with at least two supportive criteria (excellent response to levodopa, presence of levodopa-induced dyskinesia or rest tremor of a limb documented on clinical examination) without no absolute exclusion criteria or red flags. If the EHRs described parkinsonism combined with presence of up to two red flags counterbalanced by supportive criteria and absence of absolute exclusion criteria, we classified participants as possible PD. Participants without EHRs were excluded since this would require review of Paper Health Records in at least two, sometimes three hospitals in the region, which we considered too laborious. Main outcome measure was the accuracy of the clinical diagnosis of PD for participants in the HUNT study.

This study was approved by the Ethics Committee of Central Norway, and informed consent was obtained from all participants.

## Results

**Fig. 1:**
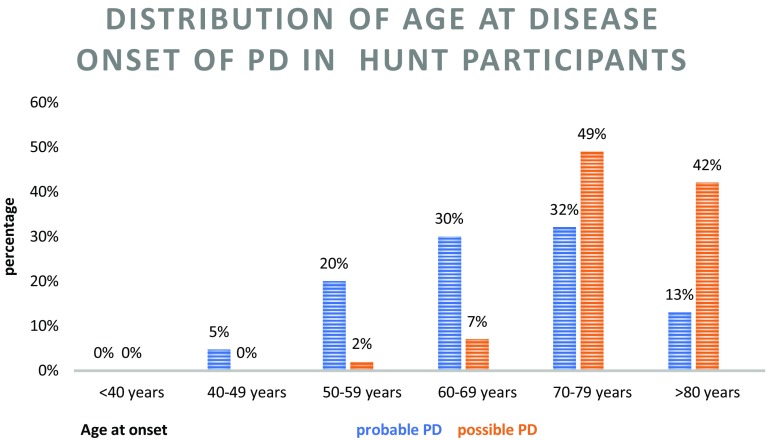
The distribution of Age at disease onset (AAO) of the participants with verified PD

In total, we identified 980 participants in the HUNT study who had been diagnosed with parkinsonism. However, we excluded 39% (379/980) due to lack of EHRs. Participants in the HUNT1 Survey only, accounted for the majority of those (75%, 263/349) as shown in Table 1. Of the participants with EHRs, 92% (550/601) had been diagnosed with PD G20 (ICD 10) /332 (ICD 9) while 8% (51/601) had been diagnosed with secondary parkinsonism G 21.1, G 21.2, G 21.3, G 21.8, G 21.9 or related parkinsonian disorders G 23.1, G 23. 9. We verified PD (probable PD and possible PD) in 65% (358/550) and excluded PD (not PD) in 35% (192/550), as shown in Table 2. Among the participants we classified as not PD, we found a related parkinsonian disorder and secondary parkinsonism distribution as shown in Table 3. Figure 1 illustrating the distribution of age at disease onset, shows predominantly older age at disease onset in the “possible PD” group compared to the “probable PD” group. The majority of participants, 71% (428/601), had been diagnosed by a neurologist, and only a few 8% (49/601) by a movement disorder expert. About 21% (124/601) had been diagnosed during hospitalization in medical, surgical or psychiatric ward. In 8% we lack information about where the diagnosis was set.

**Table 1:** The distribution of the total number of participants versus the number of participants with EHRs in the different HUNT surveys

HUNT survey	Number of participants	Number of participants with EHRs
HUNT1 survey	349	86
HUNT2 survey	26	22
HUNT3 survey	12	12
HUNT1 survey and HUNT2 survey	395	284
HUNT1 survey and HUNT3 survey	13	13
HUNT2 survey and HUNT3 survey	6	6
HUNT1 survey, HUNT2 survey and HUNT3 survey	179	178
Sum	980*	601

*PD diagnosed: G20**/**332, G21.1, G21.2, G21.3, G21.8, G21.9, G23, G23.1, G23.9

**Table 2:** The distribution of “Probable PD”, “Possible PD” and “not PD” after diagnosis validation, based on the MDS Clinical Diagnostic Criteria for PD, of EHRs of the participants who had been diagnosed G20/332 in hospitals in Trøndelag

HUNT participation	Validated EHRs of participants who had been diagnosed G20/332	Probable PD	Possible PD	Not PD
HUNT1 survey	77	35	9	33
HUNT2 survey	21	13	1	7
HUNT3 survey	12	7	1	4
HUNT1 survey and HUNT2 survey	257	139	34	84
HUNT1 survey and HUNT3 survey	13	8	1	4
HUNT2 survey and HUNT3 survey	4	2	0	2
HUNT1 survey, HUNT2 survey and HUNT3 survey	166	107	1	58
Sum	550	311	47	192

*EHR* electronic health record

**Table 3:** The distribution of related parkinsonian disorders/secondary parkinsonism among participants in HUNT in whom the PD diagnosis was excluded

Related parkinsonian disorders/secondary parkinsonism	HUNT1 survey	HUNT2 survey	HUNT3 survey	HUNT1 survey and HUNT2 survey	HUNT1 survey and HUNT3 survey	HUNT2 survey and HUNT3 survey	HUNT1 survey, HUNT2 survey and HUNT3 survey	Sum/percentage (%)
VaE	5	2	0	32	0	0	8	47 (20)
DIP	1	1	0	7	0	1	5	15 (6)
MSA	0	0	0	4	0	0	0	4 (2)
PSP	1	0	0	2	0	0	0	3 (1)
DLB	1	0	0	15	0	0	3	19 (8)
AD	4	1	0	13	0	0	1	19 (8)
Bipolar disease	2	0	0	3	0	0	4	9 (4)
Anxiety/depression	2	0	0	3	0	0	5	10 (4)
ET	2	0	1	6	0	0	7	16 (7)
Tremor	2	2	1	1	0	0	9	15 (6)
Dementia unspecified	6	1	0	10	0	0	1	18/(7)
Parkinsonism unspecified	2	0	0	4	0	0	1	7 (3)
Others	7	0	1	3	4	1	21	37 (15)
Coder errors	6	1	1	6	0	2	5	21 (9)
Total	41	8	4	109	4	4	70	240 (100)

Others include: Schizophrenia(6), MS(5), Restless legs(1), Laryngitis(1), Ankylosing spondylitis(1), cerebrovascular disease(2), NPH = normal pressure hydrocephalus(1), colon irritabile(1), astma(1) Gastric bypass(1), Polyneuropathy(3), Inclusion body myositis (1) Corticobasal degeneration (1) ALS = amyotrophic lateral sclerosis (1), Cancer mammae (1), Spastic dysphonia(1), RA = Rheumatoid Arhtritis (1), LGMD = Limb Girdle Muscular Dystrophy(1), Cervikobrachialgia (1), Claudication(1), Ca.pancreatis (1), COPD = chronic obstructive pulmonary disease(1), SWEDD = Scans without evidence for dopaminergic deficit (1), Head injury(1), Torticollis(1)

*VaE* vascular encephalopathy, *DIP* drug induced parkinsonism, *MSA* multiple system atrophy, *PSP* progressive supranuclear palsy, *DLB* dementia with Lewy bodies, *AD* Alzheimer disease, *ET* essential tremor

## Discussion

The aims of our study were to assess the accuracy of the clinical diagnosis of PD for participants in the HUNT study. This was a diagnosis validation study, as opposed to an audit of coding practices. The diagnostic accuracy in our study, 65%, is consistent with the results previously reported by Rizzo et al. [2] who evaluated diagnostic accuracy of clinical diagnosis of PD, reported in the last 25 years by a systematic review and meta-analysis. According to their results, the accuracy was 73.8% for clinical diagnosis performed mainly by non-experts and 79.6% for clinical diagnosis performed by movement disorder experts, rising from 79.6% of initial assessment to 83.9% of refined diagnosis after follow-up. Mean disease duration at last evaluation was 10.2 years, with range from 3.6 to 13.8 years [2].

There are numerous possibilities for incorrect diagnostic coding both in the “patient trajectory” and the ‘‘paper trail’’ [4]. One important source of error is the misdiagnosis made by the physicians. Low diagnostic accuracy is particularly relevant in the earliest stages of parkinsonism and presumably in older patients, even for movement disorder experts. This is due to the absence of hallmarks of atypical parkinsonism and less defined response to dopaminergic treatment [2]. Figure 1, illustrating the distribution of age at disease onset in HUNT participants, shows predominantly older age at disease onset in the “possible PD” group compared to the “probable PD” group.

The clinical diagnosis of PD was revised in a number of patients at follow-up due to lack of observed efficacy of levodopa or development of additional atypical features. However, the misdiagnosis was still present in the EHRs, which may cause incorrect selection of patients when performing diagnosis specific search in the registries. Rizzo et al. [2] reports a disease duration ranging from 3.6 to 13.8 years (mean 10.2 years) as useful to increase the diagnostic accuracy, without a clear trend of improvement of the accuracy over time. In trials, incorrect encoding may cause recruitment of participants without the trial specific diagnosis and exclusion of candidates. This may be a challenge, particularly in clinical trials designed to recruit participants with early PD [2].

Both unintentional and intentional coder errors, such as misspecification, unbundling, and up-coding, are additional potential sources of errors [4]. Since we have limited knowledge about the diagnostic coding process in hospitals in Trøndelag, we cannot estimate the level of intentional or unintentional coder errors that may have occurred. However, based on a clear discrepancy between the diagnosis registered in the contact section of the EHRs and the one posed and recorded by the physician, cf. medical journal text, we found 9% (21/240) coder errors [8].

We consider lack of access to medical records for all participants as well as diagnostic validation based on EHRs of widely varying quality a limitation of our study [9, 10]. However, we have no indication that participants in the HUNT1 Survey only, were diagnosed with higher accuracy than those participating in the later HUNT surveys. Among participants with verified PD, the rate of participation in the HUNT surveys decreased as the disease progressed causing participation (response) bias and/or self-selection bias [11].

The HUNT study has several strengths. It is covering a total population aged 20–100 years within a geographical area, which includes inland and coastal municipalities with varying characteristics. Since the age range is wide, groups of people with different cohort exposures are covered. The databank includes data on an extensive range of topics (> 6000 variables) where all data are linked to the unique PIN, which enables linkage of data for each of the participants and linkage to numbers of local, regional and national health registers, with nearly complete follow-up data [12]. The HUNT study has several advantages for epidemiologic and genetic research including cost efficiency, the large amounts of available clinical data, and the ability to analyze data over time [13]. Electronic phenotypes extracted from EHRs and other registries can be paired with genetic data to identify genes underlying common human diseases [14]. However, diagnostic accuracy is crucial when combining clinical data from health information databases to the genetic data.

This study demonstrates that the overall quality of the clinical diagnosis of PD for participants in the HUNT study is not optimal. The ICD codes entered into health registers must be quality-assured before biological material obtained from these populations can be used in the search for new biomarkers for PD. Incorrect diagnosis of cases and controls may give biased effect size estimates and misleading association results.
